# Study on the Analysis of Gender Trends Among the First Authors of Publications on Budd-Chiari Syndrome

**DOI:** 10.7759/cureus.63458

**Published:** 2024-06-29

**Authors:** Marina Samuel B, Aditya Parag Chitnavis, Rajesh Yadavalli, Sai Kiran Attuluri, Keval Thakkar

**Affiliations:** 1 Biological Sciences, Kean University, Union, USA; 2 Internal Medicine, Government Medical College, Miraj, Mumbai, IND; 3 Internal Medicine, Rajiv Gandhi Institute of Medical Sciences, Adilabad, IND; 4 Internal Medicine, Konaseema Institute of Medical Sciences and Research Foundation (KIMS&RF), Amalapuram, IND; 5 Internal and Hospital Medicine, Moffitt Cancer Center, Tampa, USA; 6 Internal Medicine, Larkin Community Hospital, Division of Research and Academic Affairs, South Miami, USA; 7 Transplant Research, Georgetown University, District of Columbia, USA

**Keywords:** statistical model, disparities, authorship, gender trends, budd-chiari syndrome

## Abstract

Introduction: Budd-Chiari syndrome (BCS) is primarily a disease of hepatic vein blockage, which involves a backflow of blood to the liver. Although there have been many causes linked to this disease, most commonly, it occurs due to hypercoagulable states and blood disorders. In recent times, there has been a fast spread of knowledge regarding early diagnosis and various treatment modalities, which has enabled the prevention of mortality in most cases. This has primarily spread through research articles published in various journals. Thus, the article aims to compare the gender trend ratios to identify the associated discrepancies in terms of male and female author contributions who have been the primary authors for articles pertaining to this disease.

Methodology: A PubMed database between the years 2013 and 2022 was used for the bibliometric analysis. The gender of the primary author was analyzed by NamSor, an application programming interface (API). The statistical analysis was conducted using R software, the ARIMA model, and graphs were prepared using Datawrapper.

Results: Out of 667 articles extracted, the analysis showed that there were 455 (68.2%) first male authors and 212 (31.8%) first female authors. We also formulated various other results, which depicted a higher female-to-male author ratio including various journals and different countries. Although there has been an increasing trend of male authors as compared to female authors, this study found that male authorship for research on this disease is still higher.

Conclusions: This study depicts that there is a necessity to draw attention to the inequitable systems favoring men over women for publications. The predictive analysis conducted also helps to foresee the trend in the next few years and explains the necessity of addressing the disparities among both genders in healthcare systems.

## Introduction

Budd-Chiari syndrome (BCS) is a condition that occurs when there is hepatic outflow obstruction in the absence of right heart failure or constrictive pericarditis. Acute BCS is uncommon and clinically characterized by ascites, hepatomegaly, and hepatic insufficiency. In most cases, patients present with chronic BCS and dysmorphic liver disease with variable fibrosis deposition [1]. Polycythemia vera, the postpartum state, and hepatocellular carcinoma are most often associated with hypercoagulable states. Treatment modalities aim at the prompt reversal of obstruction of hepatic drainage and include anticoagulation, surgical release of obstruction, or liver transplantation [2].

Gender disparities persist in the study of BCS, despite the condition's association with risk factors, such as oral contraceptive use and pregnancy, that predominantly affect women. Representation of females in BCS research, clinical cases, and literature remains varied, highlighting the need for further investigation into these disparities [3,4,5]. Furthermore, the number of female gastroenterologists and fellows is significantly lower than in other specialties for the past decade [6]. Female gastroenterologists also face gender biases in terms of pay, workload, and training opportunities [7,8].

In recent decades, significant advancements in diagnostic imaging and therapeutic strategies have improved early detection and treatment outcomes for BCS, highlighting the critical role of academic publications in disseminating these advancements. However, despite these strides, disparities persist in authorship within the medical literature, with men historically outnumbering women in publications across various medical specialties [9]. These disparities may stem from unequal opportunities, funding discrepancies, differing career trajectories, and additional responsibilities outside academia [10,11].

This study aims to address these disparities by conducting a comprehensive bibliometric analysis of gender trends among first authors in BCS publications, and in doing so, this study seeks to elucidate current gender disparities and identify contributing factors within the field. Insights gained will contribute to ongoing discussions on gender equity in medical publishing and inform strategies to promote inclusivity and diversity in research collaborations and scholarly outputs. Looking ahead, findings from this study are expected to inform future research directions and interventions aimed at fostering a more equitable environment in academic medicine, ensuring that advancements in BCS and other specialties benefit from diverse perspectives and contributions.

Aims and objectives

This study aimed to analyze the gender trends of the first author among publications on BCS from 2013 to 2022 according to country and year. By identifying and understanding the underlying reasons for gender disparities in authorship, this study aims to raise awareness of existing inequalities, promote gender equity in academic publishing, and provide actionable recommendations for fostering inclusivity and diversity in research collaborations within the field of hepatology and vascular medicine.

## Materials and methods

Study design and data collection

The analysis of bibliographic data focused on articles related to BCS, which were obtained exclusively from the PubMed database on May 9, 2023. PubMed was selected for its extensive collection of peer-reviewed literature and free accessibility, ensuring widespread dissemination of the results. The systematic search employed the term "Budd-Chiari syndrome" with a publication date range of January 1, 2018, to December 31, 2022, to emphasize recent literature. All case reports, research articles, and review articles were included in the study. All results were sorted chronologically, and the articles were downloaded as a .csv file. The authors' full names were collected, and countries of origin were extracted based on their institutional affiliations.

Gender determination

The NamSor application programming interface (API) was employed to determine the gender of the first authors and their respective country affiliations using a probabilistic approach based on an individual's full name and country of origin [12]. The authors' country affiliations were deduced from their institutional affiliations. Previous studies, for instance, those by Kuruvila et al. and Bernardi et al., have validated the reliability of the NamSor API for author gender determination [9,13]. The API assigns a probability range (0-1) for an individual's gender. Authors with a probability of 0.6 or lower underwent manual verification against their public institutional profiles, and any discrepancies were corrected by the researchers. Authors whose gender remained unidentified were excluded from the analysis.

Data analysis

The acquired data were organized and initially processed in Microsoft Excel (Microsoft Corporation, USA). Statistical analysis was carried out using RStudio (version 4.3.1, RStudio Team, RStudio: Integrated Development for R, RStudio, PBC, Boston, MA), with Fisher's exact test applied to investigate the relationship between the variables of gender and country. An autoregressive integrated moving average (ARIMA) model was used for a time-series analysis in order to forecast future data trends. Data visualization was achieved with the help of Datawrapper.

## Results

In this study, a total of 667 published articles associated with BCS were analyzed. All published articles contained the full first name of the first author. Out of 667 articles analyzed, it was found that the majority of the first authors were male (n = 455; 68.22%), while female authors were only 212 (31.78%).

**Figure 1:**
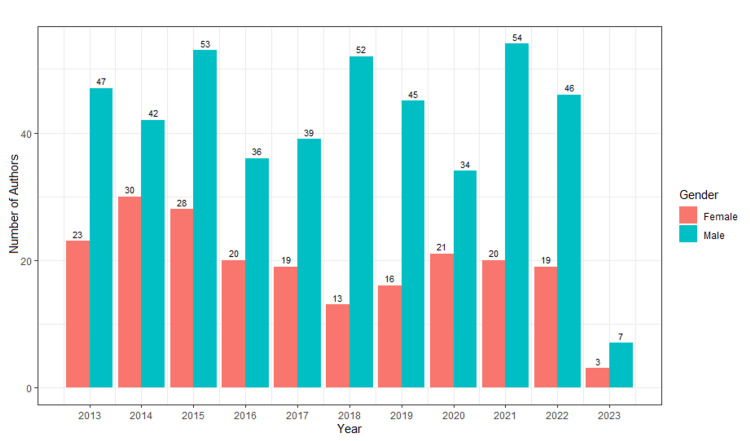
Total number of male and female authors per year

Figure 1 shows the total number of male and female first authors based on year. The maximum number of female first authors in BCS publications was 30 (41.7%) in 2014, as compared to 42 (58.3%) male first authors in the same year.

**Figure 2:**
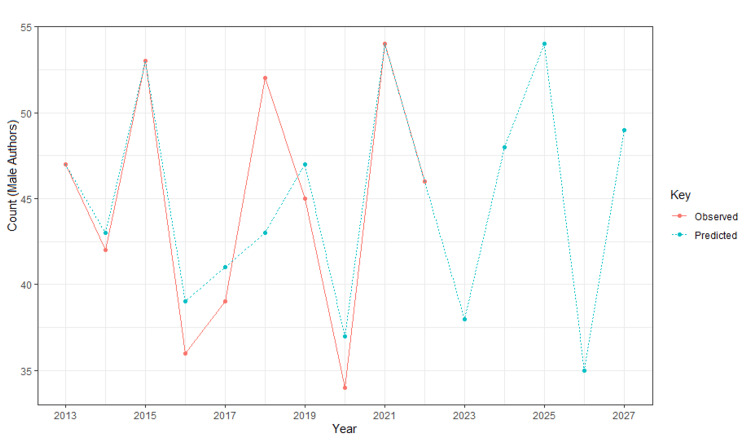
Publication trends among male first authors in Budd-Chiari syndrome research Observed trends (red): between 2013 and 2022. Predicted trend (blue): between 2023 and 2027.

**Figure 3:**
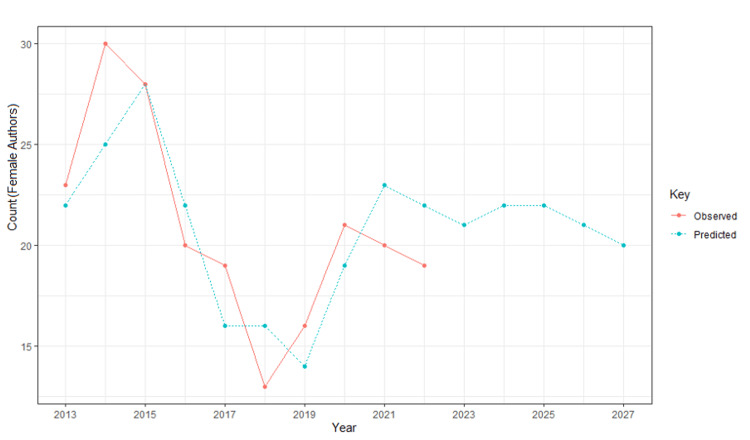
Publication trends among female first authors in Budd-Chiari syndrome research Observed trends (red): between 2013 and 2022. Predicted trends (blue): between 2023 and 2027.

Figure 2 and Figure 3 depict the trends among male and female first authors; the red line indicates the observed trends between 2013 and 2022, and the blue indicates the predicted trends from 2023 and 2027. Figure 2 shows a fluctuating trend in the number of male first authors between 2013 and 2022 and continuing fluctuation in the numbers between 2023 and 2027. Figure 3 shows an increase in the number of female first authors from 2013 to 2014, followed by a decrease in numbers until 2018. From 2018 to 2020, there is an incline in numbers, followed by a decline from 2020 to 2022. The predictive analysis forecasts a comparatively increased number of female authors but with a slight decline from 2025 to 2027.

**Figure 4:**
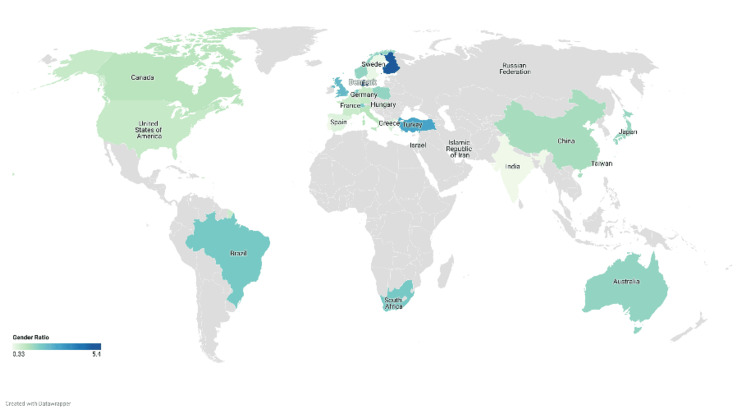
Gender trends in Budd-Chiari syndrome publications (2013-2023) based on the country and geographic distribution of the first authors.

Figure 4 illustrates the gender ratio in various countries, based on the geographic distribution of the first authors. Sweden has the highest women-to-men ratio (5.4), while India has the lowest (0.33).

Table 1 shows the top 10 journals with more than five publications and having a high sex ratio. The *Journal of Hepatology* had the highest female-to-male first-author ratio (2.5), followed by the *Abdominal Radiology Journal*, with a ratio of 1.33.

**Table 1:** Top 10 journals, with more than five publications in total, having a high gender ratio.

Journal/book	Female	Male	Ratio
J Hepatol	5	2	2.5
Abdom Radiol (NY)	4	3	1.33
Clin Res Hepatol Gastroenterol	4	6	0.67
BMJ Case Rep	5	8	0.62
Cardiovasc Intervent Radiol	3	5	0.6
Exp Ther Med	4	7	0.57
Eur J Gastroenterol Hepatol	6	12	0.5
Medicine (Baltimore)	3	6	0.5
J Gastroenterol Hepatol	4	9	0.44
Zhonghua Gan Zang Bing Za Zhi	2	5	0.4

Table 2 highlights the top 10 countries with a significant female-to-male first-author ratio. Spain has the highest ratio (1.75), followed by Pakistan (1.33).

**Table 2:** Top 10 countries having a high female-to-male first-author ratio (with more than five publications).

Country of the first author	Female	Male	Ratio
Spain	7	4	1.75
Pakistan	4	3	1.33
Portugal	5	4	1.25
France	10	10	1.00
Germany	5	5	1.00
Korea	3	3	1.00
Romania	4	4	1.00
China	80	137	0.58
USA	18	34	0.53
United Kingdom	7	15	0.47

Table 3 highlights the top 10 female first authors with the highest number of publications. X Qi has the highest number of publications as a female first author (17), followed by Andrea Mancuso with a total of six publications.

**Table 3:** Top 10 female first authors with the most publications out of the analyzed articles (with more than five publications)

First author	Full name of the first author	Country of the first author	Institution	Publications
Qi X	X Qi	China	Xijing Hospital of Digestive Diseases, Fourth Military Medical University	17
Mancuso A	Andrea Mancuso	Italy	Centro di Riferimento Regionale Malattie Rare, Sindrome di Budd-Chiari e Teleangectasia Emorragica Ereditaria, Medicina Interna 1, ARNAS Civico	6
Van Wettere M	Morgane Van Wettere	France	University Hospitals Paris Nord Val de Seine, Beaujon, Clichy	3
Su L	Lei Su	China	First Affiliated Hospital of Zhengzhou University, Zhengzhou, Henan Province	3
Zhou PL	Peng-Li Zhou	China	The First Affiliated Hospital, Zhengzhou University, Zhengzhou, Henan	3
Dang X	Xiaowei Dang	China	the First Affiliated Hospital of Zhengzhou University	3
Fu YF	Yu-Fei Fu	China	Xuzhou Central Hospital, 199 South Jie-fang Road, Xuzhou	3
Li X	Xiao Li	China	Pediatric Heart Center, West China Hospital, Sichuan University	2
Zhang Y	Yu Zhang	China	The Chinese University of Hong Kong, Prince of Wales Hospital	2
Wang Q	Qiuhe Wang	China	National Clinical Research Centre for Digestive Diseases and Xijing Hospital of Digestive Diseases, Fourth Military Medical University	2

## Discussion

This study yielded several interesting results. It was found that the gender ratio of first-author publications on BCS was 17:8 (male:female). Hence, from this study, it can be observed that female authorship continues to trail behind male authorship in BCS. These findings are supported by the study by Rickard et al., who analyzed 750 urology papers between 1990 and 2019, of which 70% of the first authors were male [14]. Another study by Baobeid et al. also discovered that among the articles published in the year 2014-2016 in Africa, 61% of the first authors were male [15]. Moreover, a study conducted by Morgan et al. revealed that between 2013 and 2018,* The* *Lancet Global Health* published a total of 1,323 articles, and of the 5,878 authors who contributed to the publications, only 2,020 (34%) were women [16].

Examining gender trends over time revealed a noteworthy surge in publications authored by women in 2022, which could be attributed to a positive trend toward increased representation and engagement of female researchers in BCS research. Moreover, our forecasting model anticipates a continued upward trajectory in publications by both male and female authors in forthcoming years. This aligns with the findings of Kuruvila et al. and Bernardi et al., who observed a narrowing gender gap and enhanced female participation in publication trends [9,13]. The trends could also be due to geographical variations; according to a 2018 report on the indicators of female contributions to scientific publications, the United States ranks in the middle of the top 50 highest-publishing countries in terms of female scientific authors [17]. In spite of this progress, a significant gender gap in authorship in specialist fields such as hand surgery and otolaryngology still persists [18,19].

Inequitable systems continue to be imposed on women in the field of global health, which influences gender inequalities in academic publications. For example, women are less likely to receive financial grants, restricting their ability to conduct and publish research [16,20]. The present study also noted a significant male-to-female ratio evident across various journals, suggesting potential systemic issues within academic publishing. While journals with rigorous acceptance criteria and blinded peer review processes strive for high standards and reduced gender bias, they may inadvertently create barriers to achieving publication for certain groups, particularly women [21,22]. These disparities emphasize the importance of addressing gender imbalances and fostering greater opportunities for female researchers in BCS-related studies. Such efforts are essential to ensure equitable contributions to BCS knowledge and to promote inclusivity within academic discourse. 

It is essential to draw attention to unfair systems and practices that fail to support female authors. To increase authoring opportunities, they must compromise between the need to publish and the utilization of their own position and influence. To enhance diversity in global health publications and ensure that female writers have equal opportunities for growth, it is essential to address such disparities. The inclusion of a table highlighting the top 10 female authors with the highest publication counts underscores the significant contributions of women in advancing BCS-related literature, with X Qi having the highest number of publications as the first author.

Limitations

Data were extracted from the PubMed database only; other electronic databases were not used. Only the first author was included with the assumption that they would most likely initiate and lead the study. There are a limited number of studies available on BCS in the chosen time frame. The software program NamSor cannot be relied upon completely because of its improvising algorithms. A few author names were excluded from the data because of the unavailability of their complete names.

## Conclusions

In summary, our study underscores a notable gender disparity in research publications on BCS, with men representing the majority of authors compared to women. Analyzing data spanning 2013 to 2022, we observed a declining percentage of female researchers contributing to the literature over time. While 2014 marked a peak in female representation, subsequent years showed a persistent trend toward fewer female-authored publications. Our findings underscore the importance of recognizing and addressing gender imbalances within academic publishing, particularly in fields such as BCS research, to ensure diverse perspectives and equitable contributions to scientific knowledge.

Moving forward, it is imperative to explore the underlying factors contributing to the observed gender disparity and implement strategies aimed at fostering greater gender diversity in research publications. This may involve initiatives to support and encourage female researchers, such as mentorship programs, funding opportunities, and policies that promote inclusivity in academic environments. In addition, future research should expand beyond first-author analysis to include all authors, providing a more comprehensive understanding of gender trends in BCS research. By promoting gender equity and inclusivity in scientific publishing, we can enhance the quality and breadth of research in BCS and other fields, ultimately advancing our understanding and management of this complex condition.
